# Intracranial pressure, lateral sinus patency, and jugular ultrasound hemodynamics in patients with venous pulsatile tinnitus

**DOI:** 10.3389/fneur.2022.992416

**Published:** 2022-09-16

**Authors:** Xiuli Gao, Yue-Lin Hsieh, Shenjiang Wang, Suming Shi, Wuqing Wang

**Affiliations:** ^1^Department of Radiology, Eye Ear Nose & Throat Hospital, Fudan University, Shanghai, China; ^2^Department of Otology and Skull Base Surgery, Eye Ear Nose & Throat Hospital, Fudan University, Shanghai, China; ^3^NHC Key Laboratory of Hearing Medicine, Shanghai, China

**Keywords:** pulsatile tinnitus, cerebrospinal fluid pressure, intracranial pressure, idiopathic intracranial hypertension, transverse sinus stenosis, jugular hemodynamics, sinus hemodynamics, arachnoid granulation

## Abstract

The clinical and hemodynamic characteristics of venous pulsatile tinnitus (PT) patients with normal or elevated cerebrospinal fluid pressure (CSFP) have not been clearly differentiated. This study aimed to explore CSFP among patients with PT as the solitary symptom, as well as quantitatively and qualitatively assess the role of the degree of transverse sinus (TS) stenosis and jugular hemodynamics in venous PT patients. A total of 50 subjects with venous PT with or without sigmoid sinus wall anomalies (SSWAs) were enrolled in this study. In addition to radiologic assessments for TS stenosis and invagination of arachnoid granulation (AG) in TS, CSFP and jugular hemodynamics were measured *via* cerebrospinal fluid (CSF) manometry and Doppler ultrasound. Apart from group comparisons and correlation analyses, multivariate linear regression, and receiver operating characteristic (ROC) models were used to identify the sensitivity and specificity of the index of transverse sinus stenosis (ITSS) and hemodynamic variables with inferential significance. The mean CSFP of all cases was 199.5 ± 52.7 mmH_2_O, with no statistical difference in CSFP between the diverticulum and dehiscence groups. Multivariate linear regression analysis demonstrated that CSFP was linearly correlated with ITSS and pulsatility index (PI). ROC analysis showed that the area under the ROC curve of PI was 0.693 at 200 mmH_2_O threshold, and the best PI cut-off value was 0.467, with a sensitivity of 65.7% and specificity of 81.8%. For 250 mmH_2_O threshold, the area under the ROC curve of PI was 0.718, and the best PI cut-off value was 0.467 with a sensitivity of 68.4% and specificity of 75.0%. Additionally, the area under the ROC curve of ITSS was 0.757, and the best ITSS cutoff value was 8.5 (*p* = 0.002, 95% CI = 0.616–0.898) with a sensitivity of 72.4% and specificity of 75.0% at 200 mmH_2_O threshold. In conclusion, patients with venous PT as the only presenting symptom should be suspected of having borderline or increased CSFP when they present with high ITSS, BMI and low PI. Further, AG in TS without encephalocele and empty sellae are not limiting findings for differentiating the level of CSFP in patients with venous PT.

## Introduction

Vascular pulsatile tinnitus (PT), or pulse-synchronous tinnitus, is an abnormal self-perception of rhythmic vascular somatosounds (1–3). As a type of objective tinnitus, vascular PT is categorized into arterial, arteriovenous, and venous origins (4, 5). Venous PT is the largest demographic in patients with vascular PT and is associated with a) anatomical anomalies, such as sigmoid sinus wall anomalies (SSWAs), transverse-sigmoid sinus enlargement, and transverse sinus (TS) stenosis, and b) intracranial hemodynamic abnormalities, such as increased cerebrospinal fluid pressure (CSFP), increased trans-stenotic pressure gradient of the TS, and dural venous sinus flow volume asymmetry (5–9). While these anatomical and hemodynamic anomalies synergistically produce and allow the transmittance of PT, SSWAs are recognized as the most deterministic factor that causes PT (7).

SSWAs and TS stenosis are common radiologic findings in patients with venous PT (9–12). Zhao et al. found that PT is more relevant to the presence of SSWAs than TS stenosis severity and location (12). Since SSWAs are the most common findings attributable to the cause of PT (5), neuro-otologists adopt transtemporal sinus wall reconstruction surgery to eradicate PT by addressing ipsilateral SSWAs and/or abnormal flow hemodynamics (13–17). Despite therapeutically effective, the level of CSFP in surgical candidates is usually not evaluated on a regular basis, which leaves the underlying symptoms related to idiopathic intracranial hypertension (IIH) often unidentified and renders the assessment of surgical safety and postoperative changes in CSFP unpredictable.

IIH is a disorder characterized by abnormally increased CSFP for unknown reasons. The age-and sex-adjusted annual incidence of IIH was reported to be 0.9 per 100,000 persons and 3.5 per 100,000 in females 15–44 years of age, predominantly affecting obese women of childbearing age (18, 19). Common symptoms of IIH include headache (76–94%), transient visual obscurations (68–72%), pulsatile tinnitus (PT) (52–61%), and neck and back pains (42–53%) (20–22). Common radiologic findings include TS stenosis, empty sellae, flattening of the posterior globes, optic nerve sheath distension, and brain herniation, but none of these findings are pathognomonic (21–24). However, up to 93% of the subjects presented with unilateral or bilateral TS stenoses (25). Morris et al. suggested that the degree of bilateral TS stenosis is a more sensitive indicator than other radiologic (e.g., empty sellae) and ophthalmologic findings (e.g., papilledema) (25). An increasing number of neuro-radiologic studies have suggested that TS stenting successfully lowers CSFP with a high success rate (26). The expansion of the venous compartment after stenting lessens both the trans-stenotic pressure gradient and regional flow velocity, which diminishes CSFP and eliminates PT as a secondary benefit (27). Besides causing an increase in CSFP, TS stenosis has also been suggested to induce post-stenotic hemodynamic anomalies that may promote erosion of the sigmoid plate (28, 29).

Several anatomic variations such as arachnoid granulation (AG) and brain herniation (encephalocele) have been shown to disrupt flow patterns and/or cause intrinsic TS stenosis (12, 20). However, both anatomical variations are *post-hoc* radiological findings that make it difficult to discern the growth or innate existence of their presence. AG, originally considered a one-way valve that plays a key role in CSF drainage, has been associated with a compensatory mechanism caused by increased CSFP (30, 31). Nevertheless, AGs also prevail among normal populations, and their number, distribution, and size can vary significantly over a lifetime (32). In a recent study, Smith et al. identified 6% of PT subjects with brain herniation into the AG situating inside the TS lumen. They urged that brain herniation into AG is a common yet overlooked finding in patients with PT, making them prone to IIH (33).

Intracranial pressure has previously been monitored using pulsatility index (PI)-and cerebral perfusion pressure-based techniques *via* the transcranial Doppler method (34–36). According to the Monro-Kellie doctrine, the sum of the brain, CSF, intracranial arterial/venous blood volumes, and glymphatic system (possibly the fourth and only pathologic component within our cranial cavity based on recent growing CSF dynamic studies) is a constant (21, 37). Thin-walled venous sinuses are vulnerable to generation compression, which counteracts the increase in CSFP (38). Furthermore, a reduction in the venous component induces an increase in blood flow velocity/volume and attenuation in the pulsatile component of the venous outflow (39). These phenomena and the alteration of CSF-venous sinus hemodynamics have been reported in subjects with IIH and other IIH-associated clinical entities (40–42).

Given the close correlation between CSFP homeostasis, TS patency, and sinus flow hemodynamics, quantitative and qualitative assessment of CSFP by evaluating the degree of TS stenosis and the pulsatile component of venous outflow can provide a deeper insight into the potential level of CSFP in patients with venous PT. The primary goal of this study was to reveal the prevalence and numeric correlation among the levels of CSFP, TS stenosis, and jugular outflow hemodynamics among patients with venous PT as a solitary symptom. Evaluation of CSFP in patients with venous PT can be decisive for customizing transtemporal surgical strategies to reduce the risk of iatrogenic cause of intracranial hypertension. The secondary goal of this study was to unveil a linear correlation among physical/sinus morphologies, hydrodynamics, and CSFP to provide a basic understanding of related factors of CSFP.

## Materials and methods

### Participant data and study design

In this retrospective study, we enrolled 50 venous PT patients who were treated at the Otology and Skull Base Surgery Center of the Eye, Ear, Nose, and Throat Hospital at Fudan University from December 2018 to July 2022. The diagnostic criteria for venous PT included: 1) changes in perception of PT (reduction and/or disappearance of PT) *via* ipsilateral internal jugular vein (IJV) compression as the essential condition and 2) with or without radiologic presentation of SSWAs. Computed tomography (CT) and magnetic resonance (MR) angiogram/venogram were performed during the initial clinical evaluation to exclude patients whose PT was not related to venous etiologies, such as arterial and arteriovenous vascular origins, systemic diseases such as anemia and hyperthyroidism, and PT caused by temporal bone neoplasms and third mobile window syndrome. Finally, lumbar puncture was performed to reveal CSFP in participants.

This study primarily focused on the numerical correlation and linearity of normalized sinus morphologic data and the value of CSFP. Additionally, participants were subdivided into (1) <200 and ≥200 mm H_2_O to juxtapose those with or without elevation of CSFP (detailed definitions are provided in the section 2.4) and (2) diverticulum, dehiscence, and non-SSWA case groups to examine the impact of CSFP on sigmoid sinus wall. A diverticulum was defined as the protrusion of an irregularly shaped sigmoid sinus vascular wall into the mastoid cortex and/or air cells. Dehiscence was defined as the absence of a bony plate overlying the sigmoid sinus vascular wall, and non-SSWAs were defined as subjects with intact sigmoid plates. The dominance of the sinus system was determined by the larger laterality of the transverse-sigmoid sinus system, based on the MR venogram. Contralateral TS stenosis was defined as discontinuity of the sinus lumen and over 40% smaller TS caliber (43).

The studies involving human participants were reviewed and approved by Ethical Committees of the Eye, Ear, Nose, and Throat Hospital in Shanghai, China. The patients/participants provided their written informed consent to participate in this study.

### Measurement of TS stenosis and identification of AG

**Figure 1:**
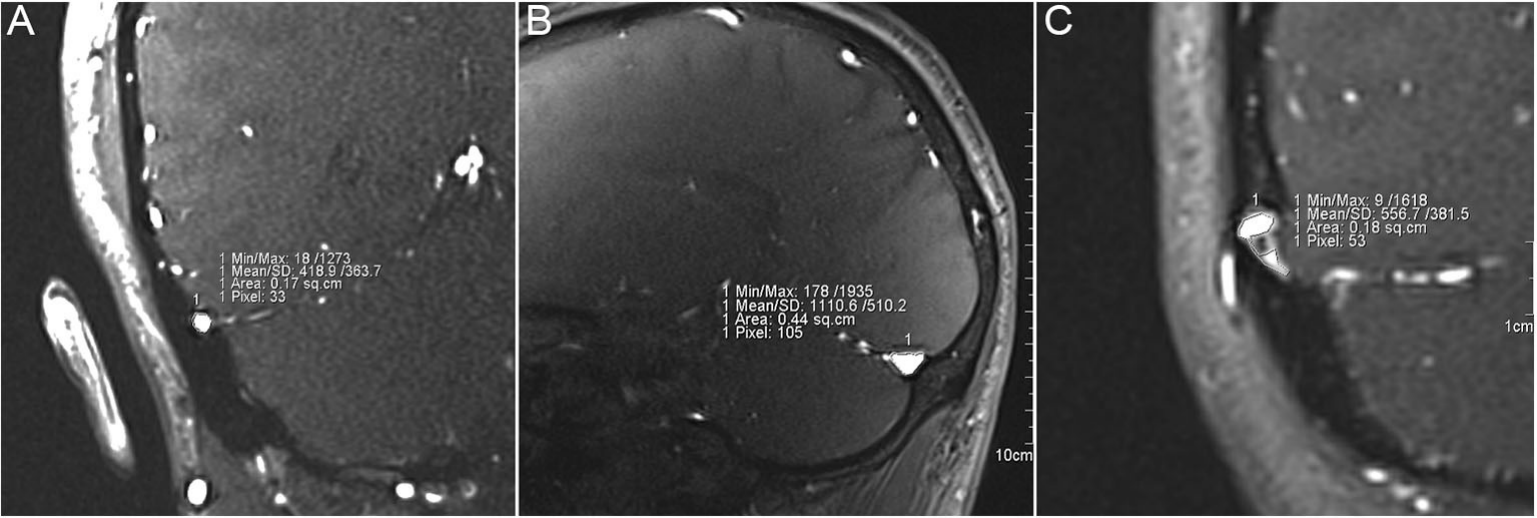
Measurement of the cross-sectional area of transverse sinus (TS) stenosis. **(A)** Measurement of the cross-sectional area from distal (transverse-sigmoid sinus junction) to middle TS lumen using coronal magnetic resonance (MR) images. **(B)** Measurement of the cross-sectional area of proximal (confluence of the sinus) to middle TS lumen using coronal MR images. **(C)** Measurement of cross-sectional area of TS lumen with endovascular invagination of arachnoid granulation.

A total of 92 TS in 46 participants were measured on the workstation NUMARIS/4 (SYNGO MRB17, Siemens AG) or software Mimics 19.0 (Materialize, Belgium) using patient-specific 2D-Time-of-Flight MR venograms (Figure 1). Four subjects without MR images were excluded from this study. All MR images were acquired from MAGNETOM Verio 3.0-T (Siemens AG, Muenchen, Germany) MR scanner. All parameters were set equivalent to our previous study (Table 1) (17). TS stenosis was determined between the proximal end (1 cm distal to the TS and superior sagittal sinus/straight sinus intersection point using sagittal MR slices) and the distal end (1 cm proximal to the TS-SS junction using coronal MR slices) of the TS. The normalized degree of TS stenosis was measured on either side of the TS and was defined as:


$$\begin{array}{l} \text{TS}{\text{S}}_{\text{Normalized}}=\frac{\text{cross}.\text{are}{\text{a}}_{\max}-\text{ cross}.\text{are}{\text{a}}_{\min}}{\text{cross}.\text{are}{\text{a}}_{\max}}, \end{array}$$

where the cross.area_max_ and cross.area_min_ represent the largest and smallest cross-sectional area of the ipsilateral TS, respectively. The index of transverse sinus stenosis (ITSS) was implemented to categorize the degree of TS stenosis based on the Carvalho criteria (44):


$$\begin{array}{l} \text{ITSS}=\text{degree of left TS stenosis }\times \text{degree of right TS stenosis}, \end{array}$$

where the degree of left/right TS stenosis was classified into five disparate scales: 0 indicated normal, 1 indicated TS stenosis <33%, 2 indicated TS stenosis 33–66%, 3 indicated TS stenosis >66%, and 4 indicated TS hypoplasia.

**Table 1:** Sequences and parameters of magnetic resonance (MR) imaging.

	**Sequence**	**TE/TR (ms)**	**Acquisition matrix**	**Flip angle**
T1w	t1_tse	12.0/840.0	320 × 240	150
T2w	t2_tse_fs	82.0/4660.0	384 × 307	150
T1w with contrast	t1_fl2d_fs	4.2/267.0	320 × 240	70
MRV	TOF-2D-obl	5.0/21.0	320 × 272	60

**Figure 2:**
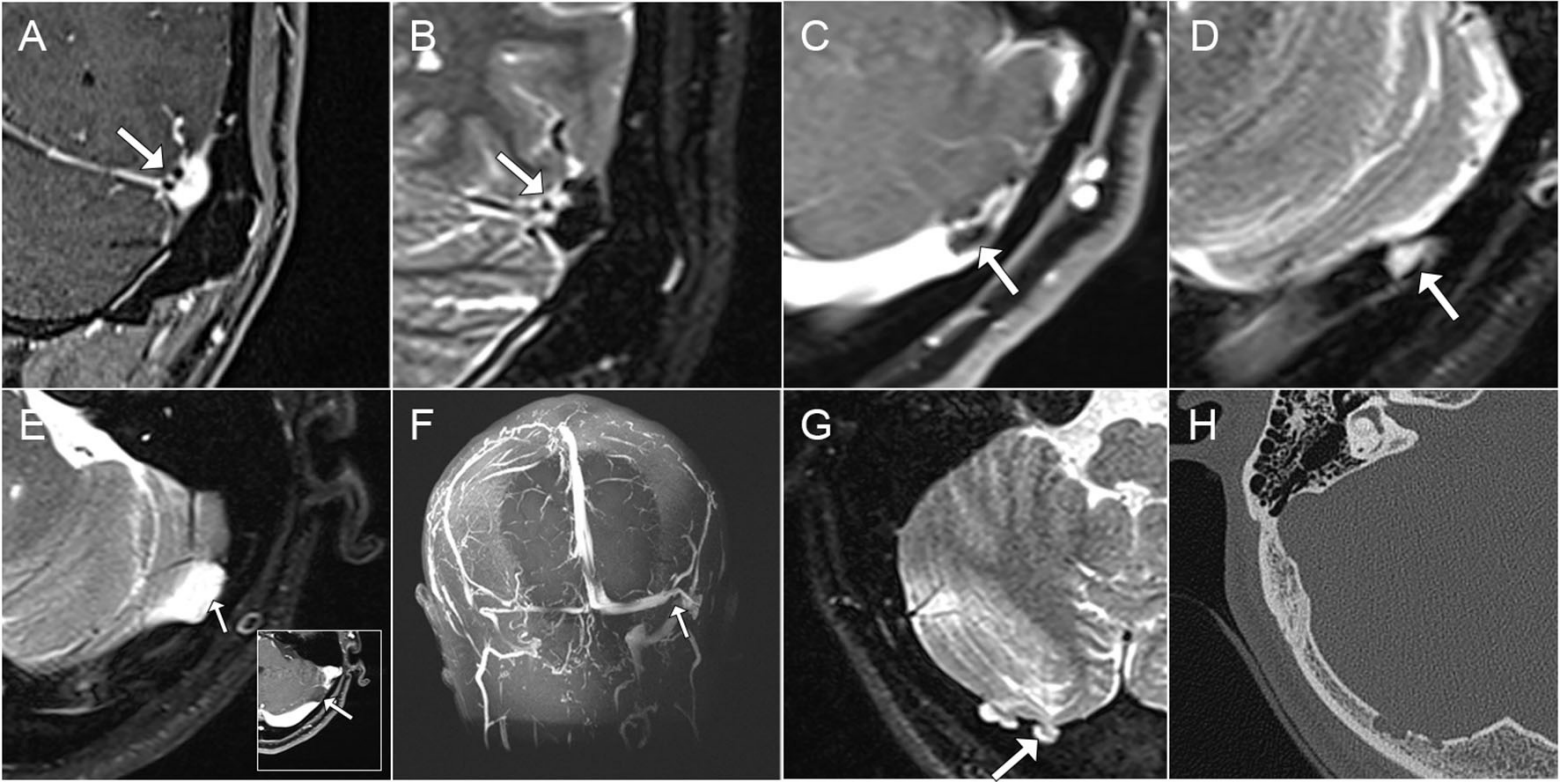
Characteristics of transverse sinus (TS) endoluminal invagination of arachnoid granulation (AG), arachnoid cyst, and brain herniation into AG. **(A,B)** Coronal contrast-enhanced T1-weighted and T2-weighted magnetic resonance (MR) images showcasing endoluminal invagination of AG inside TS. **(C,D)** Axial contrast-enhanced T1- and T2-weighted MR images showcasing obstruction of TS lumen by an AG. **(E,F)** Axial T2-weighted/contrast-enhanced T1-weighted and Coronal maximum intensity projection MR venogram images demonstrating the intrinsic compression of TS lumen from an arachnoid cyst causing TS stenosis. **(G,H)** Brain herniation in AG perforating the occipital bone structure using axial T2-weighted MR and CT images.

Endoluminal protrusion of the AG into the TS and brain herniation into the AG were examined and cross-validated using different sequences of MR. Endoluminal invasion of the AG was determined using (1) axial and/or coronal T2 section showing the protrusion of the arachnoid membrane containing white CSF signal at the intersection of cerebrum and cerebellum and (2) contrast-enhanced T1-weighted gradient echo (2D FLASH) MR and 2D time-of-flight MR venogram images displaying *in situ* black blood filling defect inside the TS lumen (Figure 2). Encephalocele was confirmed when the herniation of the brain tissue into the AG was observed.

### Jugular outflow hemodynamics

**Figure 3:**
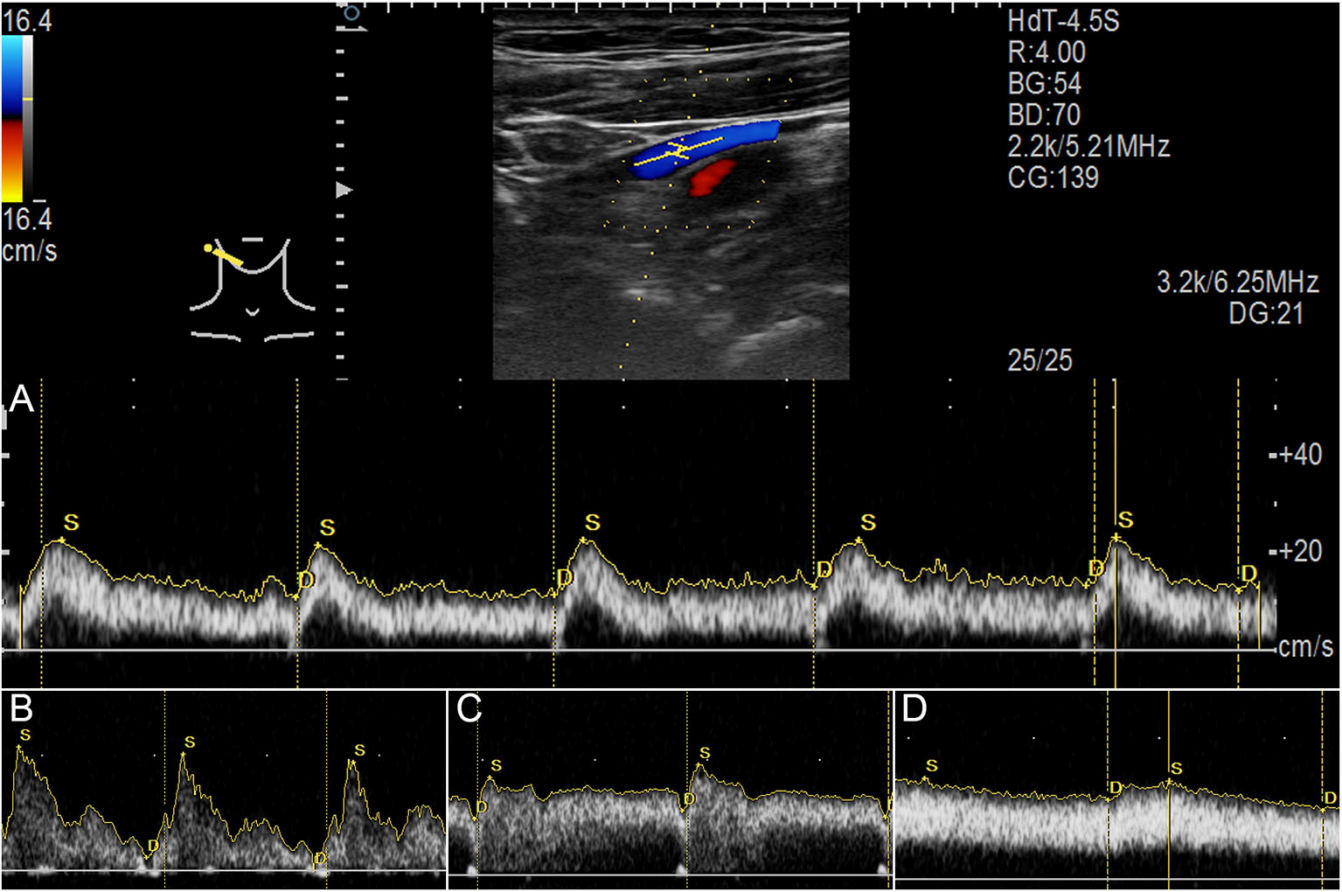
Jugular Doppler ultrasound velocity spectra. **(A)** Ultrasound measurement of upper internal jugular vein (IJV). **(B)** Flow spectrum demonstrating an averaged pulsatility index (PI) of 1.54. **(C)** Flow spectrum demonstrating an averaged PI of 0.46. **(D)** Flow spectrum demonstrating an averaged PI of 0.21.

The Doppler ultrasound system Arietta 60 ultrasound system (Hitachi Aloka Medical Ltd., Japan) with an L441 transducer (2–12 MHz) and MyLab Class C (ESOATE SpA, Genoa, Italy) with an LA-523 transducer (4–13 MHz) were used to measure real-time bilateral upper jugular outflow hemodynamics at the mandibular region and skull base. The participants were tested in a supine position with a neutral head position. The transducer was gently placed to prevent the deformation of the vascular wall. Hemodynamic parameters were measured until the morphology of the pulsating blood flow curves was stable (Figure 3). Bilateral time-averaged (at least 3.5 s) jugular outflow hemodynamic parameters, including flow volume (Vol_flow_), mean velocity (V_mn_), peak velocity (V_max_), pulsatility index (PI), and resistive index (RI), were acquired. PI and RI were defined as:


$$\begin{array}{l} \text{PI}=\;\frac{{\text{V}}_{\max}-V_{\min}}{{\text{V}}_{\text{mn}}},\\ \text{RI}=\;\frac{{\text{V}}_{\max}-{\text{V}}_{\min}}{{\text{V}}_{\max}}, \end{array}$$

where V_max_, V_mn_, and V_min_ denote the peak systolic velocity, averaged flow velocity, and minimum diastolic flow velocity, respectively. Additionally, 50 normal adult subjects without PT/subjective tinnitus, previous history of otologic diseases/symptoms, and symptoms related to elevated CSFP were recruited in this study to compare hemodynamics with the case group.

### CSF manometry

CSFP was evaluated in all participants who presented with radiologic findings highly suggestive of IIH. CSF manometry was performed in accordance with our previous methods (45, 46). Briefly, the participants were required to maintain the lateral decubitus position with their legs straightened. Open CSF pressure was recorded after the fluid level had stabilized. Since both 200-and 250-mmH_2_O thresholds were the diagnostic standard to indicate elevated CSFP (25, 47–50), this study therefore retained the use of both 200-and 250-mmH_2_O thresholds to reveal the correlation between CSFP and variables of interests.

### Statistical analysis

Statistical analysis was performed using R software (RStudio, Boston, MA, United States) and SPSS (version 20.0; SPSS Inc., Chicago, IL, United States). Statistical analysis of descriptive data was performed using Fisher's exact test. The normality of all continuous data was first checked using the Shapiro-Wilk test. The two-sample *t*-test, Mann-Whitney test, and Kruskal-Wallis test were used for statistical analysis, as appropriate. Bonferroni correction was applied to multiple tests of statistical significance on the same data. For correlation analysis, Pearson or Spearman correlation coefficient was used to calculate the normality of continuous data. The strength of the correlation was based on the Cohen criteria: very strong (0.5–1.0), moderate (0.3–0.5), or weak (<0.3) (51). Multivariate linear regression analysis was initially conducted on all continuous data using a stepwise backward elimination procedure to check the linear correlation between CSFP and correlated variables. Receiver operating characteristic (ROC) analysis was performed to identify the sensitivity and specificity for variables with inferential significance, and the optimal cut-off value was specified. Statistical significance was defined as *P* < 0.05.

## Results

### Descriptive characteristics

Among 50 participants (female:male = 44:6) with venous PT, the average age of the study population was 34.7 ± 7.9 years. In addition, 19 (38%) and 31 (62%) subjects presented with < 200 and ≥ 200 mmH_2_O CSFP, respectively, wherein CSFP ≥ 250 mmH_2_O was found in 12 (24%) subjects. The mean CSFP rate of all cases was 199.5 ± 52.7 mmH_2_O. The average body mass index (BMI) was 23.4 ± 4.1 kg/m^2^. Further, there was a significant difference in BMI between 200 mmH_2_O (Mann-Whitney test, *p* < 0.01) and 250 mmH_2_O (Mann-Whitney test, *p* = 0.045) CSFP thresholds. 27 of 46 (58.6%) subjects presented with empty sellae. The presence of empty sellae was not significantly different between 200 mmH_2_O (Fisher exact test, *p* = 0.554) and 250 mmH_2_O (Fisher exact test, *p* = 0.514) CSFP thresholds. PT reduced or subsided within 48 h in 33 (66%) subjects. Descriptive characteristics are shown in Table 2. None of subjects reported symptoms including pulsating headache, visual loss or changes in visual acuity, and neck or back pain/numbness apart from PT.

**Table 2:** Descriptive and continuous data of 50 study subjects characterized by 200-and 250-mmH_2_O cerebrospinal fluid pressure (CSFP) thresholds.

	**250 mmH_2_O CSFP threshold**	**200 mmH_2_O CSFP threshold**	***p*** **value**
	**≥250 vs. <250 mmH_2_O**	**≥200 vs. <200 mmH_2_O**	**250/200 mmH_2_O thresholds**
Number of cases	12:38	31:19	
Age (Years)	33 (31/37):35 (30/38.25)	34 (31/39):32 (30/37)	0.846^†^/ 0.206^†^
BMI (kg/m^2^)	24.6 (22.9/28.6):21.8 (20.5/23.4)	23.4 (22.4/26.9):21.2 (19.9/22.6)	0.020^†^/ <0.01^†^
Empty Sellae^#^ (Yes/No)	7/4:20/15	16/13:11/6	>0.999^*^/0.554^*^
AG^#^ (Yes/No)	6/6:14/20	11/18: 9/8	0.737^*^/0.368^*^
Diverticulum/Dehiscence/Non-SSWAs	4/7/1:20/15/3	13/15/1:11/7/3	0.537^*^/0.291^*^
ITSS^#^	9 (8/12):9 (6/9)	9 (8.5/12):6 (4/8.5)	0.230/ <0.01^†^

* Descriptive data measured using Chi-Square.

† Continuous data measured using Mann-Whitney test.

# Empty Sellae, arachnoid granulation (AG) and index of transverse sinus stenosis (ITSS) were measured on 46 participants (Increased CSFP: normal CSFP = 29: 17) using magnetic resonance images.

Variables are expressed as mean ± standard deviation or median and interquartile range.

Among the 50 subjects, there were 24 diverticulum cases, 22 dehiscence cases, and 4 non-SSWA cases. There was no significant difference between participants with and without diverticulum/dehiscence in the 200 mmH_2_O (Fisher exact test, *p* = 0.291) and 250 mmH_2_O (Fisher exact test, *p* = 0.537) thresholds. In addition, there was no statistical significance found in the value of CSFP among the three cohorts (Kruskal–Wallis test, *p* = 0.110) and between the diverticulum (195.6 ± 52.2 mmH_2_O) and dehiscent groups (212.6 ± 46.6 mmH_2_O) (two-sample *t*-test, *p* = 0.279). The average CSFP of non-SSWAs group was 152.5 ± 57.0 mmH_2_O.

### Number of AGs and degree of TS stenosis

The number of AGs and the degree of TS stenosis were measured in a total of 92 TS in 46 subjects. The complete data of the TS stenosis characteristics are shown in Tables 2, 3. In 46 participants, AG was present in 20 (43.4%), and only 4 subjects had AGs in the bilateral TS. Two patients had an arachnoid cyst that compressed the ipsilateral TS lumen. The presence of AG was not significantly different between the CSFP 200 mmH_2_O (Fisher's exact test, *p* = 0.368) and CSFP 250 mmH_2_O (Fisher's exact test, *p* = 0.737) thresholds (Table 2). In addition, the presence of AG (Fisher's exact test, *p* = 0.834) was not significantly different between the SSWAs and non-SSWA groups. Brain herniation into the AG was found in two (4%) cases whose CSFP in both cases surpassed 200 mmH_2_O, which was observed at the occipital bone below the TS lumen.

**Table 3:** Descriptive and continuous data of 50 study subjects characterized by diverticulum, dehiscence, and non-SSWAs groups.

	**Diverticulum**	**Dehiscence**	**non-SSWAs**	***p*** **value**
	**(*n* = 24)**	**(*n* = 22)**	**(*n* = 4)**	
Level of CSFP (mmH_2_O)	195.6 ± 51.0	212.6 ± 46.6	152.5 ± 57.0	0.110^†^
Empty Sellae^#^ (Yes/No)	16/6	9/11	2/2	0.157^*^
AG^#^ (Yes/No)	10/12	8/12	2/2	0.911^*^
ITSS#	9 (7.5/12)	9 (6/9.75)	4 (3.5/5)^‡^	0.038^†^

* Descriptive data measured using Chi-Square.

† Continuous data measured using Kruskal Wallis test.

‡ Statistical difference found between sigmoid sinus wall anomalies (SSWAs) vs. non-SSWAs groups, Mann-Whitney test, *p* = 0.014.

# Empty Sellae, arachnoid granulation (AG) and index of transverse sinus stenosis (ITSS) were measured on 46 participants (Diverticulum: Dehiscence: non-SSWAs = 22:20:4) using magnetic resonance venogram.

Variables are expressed as mean ± standard deviation or median and interquartile range.

Of the 46 participants, the median ipsilateral normalized degree of TS stenosis was 70.1% (53.5%/78.1%), and the contralateral normalized degree of TSS was 73.8% (63.4/78.3%), with an additional 18 (36%) subjects presenting with contralateral TS hypoplasia. The median index of transverse sinus stenosis (ITSS) scores in subjects with CSFP < 200 and ≥ 200 mmH_2_O were 6 (4/8.5) and 9 (8.5/12), respectively, which were significantly different (Mann-Whitney test, *p* < 0.01); ITSS was not significantly different in subjects between < 250 and ≥ 250 mmH_2_O CSFP thresholds (Mann-Whitney test, *p* = 0.230). It is also noteworthy that CSFP in subjects with contralateral TS hypoplasia with a median of 230 (190/257.5) mmH_2_O was significantly higher than that in subjects without contralateral TS hypoplasia, with a median of 180 (150/233.75) mmH_2_O (Mann-Whitney test, *p* = 0.037). The ITSS scores of the diverticulum, dehiscence, and non-SSWAs groups were 9 (7.5/12), 9 (6/9.75), and 4 (3.5/5), respectively, which were significantly different among the three cohorts (Kruskal Wallis test, *p* = 0.038). There was a significant difference in ITSS scores between the SSWAs and non-SSWAs groups (Mann-Whitney test, *p* = 0.014), and for intracohort comparison of ITSS, diverticulum and non-SSWAs were significantly different (Mann-Whitney test, *p* = 0.013). However, the ITSS score was not significantly different between the dehiscence and diverticulum groups (Mann-Whitney *U* test, *p* = 0.475).

### Jugular hemodynamics

The complete jugular hemodynamic data of the 50 participants and 50 controls are shown in Tables 4, 5. The bilateral-averaged PI and RI were significantly different between the 200 mmH_2_O (bilateral-averaged PI: < 200 mmH_2_O vs. ≥ 200 mmH_2_O CSFP groups = 0.88 (0.59/1.16) vs. 0.44 (0.29/0.82), Mann-Whitney test, *p* = 0.037; bilateral-averaged RI: < 200 mmH_2_O vs. ≥ 200 mmH_2_O CSFP groups = 0.54 (0.38/0.67) vs. 0.33 (0.22/0.53), Mann-Whitney test, *p* = 0.012) and 250 mmH_2_O (bilateral-averaged PI: <250 mmH_2_O vs. ≥ 250 mmH_2_O CSFP groups = 0.80 (0.42/1.14) vs. 0.34 (0.24/0.52), Mann-Whitney test, *p* = 0.024; bilateral-averaged RI: < 250 mmH_2_O vs. ≥ 250 mmH_2_O CSFP groups = 0.50 (0.32/0.66) vs. 0.26 (0.19/0.38), Mann-Whitney test, *p* = 0.037) thresholds. In the SSWAs and non-SSWA groups, only the bilateral-averaged PI and RI were found to be significantly different (bilateral-averaged PI: Kruskal Wallis test, *p* = 0.026; bilateral-averaged PI: Kruskal Wallis test, *p* = 0.035).

**Table 4:** Jugular hemodynamics of 50 study subjects are characterized by 200-and 250-mmH_2_O cerebrospinal fluid pressure (CSFP) thresholds and diverticulum, dehiscence, and non-SSWAs groups.

**Groups**	**Number of cases**	**Bilateral total Vol_flow_**	**Bilateral averaged V_mn_**	**Bilateral averaged V_max_**	**Bilateral averaged PI**	**Bilateral averaged RI**
		**g/s**	**cm/s**	**cm/s**	**–**	**–**
CSFP < 200 mmH_2_O	19	12.9 (5.8/25.0)	19.1 (11.0/27.4)	25.8 (23.4/33.2)	0.88 (0.59/1.16)	0.54 (0.38/0.67)
CSFP ≥ 200 mmH_2_O	31	18.6 (13.4/26.6)	25.5 (21.0/29.7)	34.1 (27.3/38.7)	0.44 (0.29/0.82)	0.33 (0.22/0.53)
^†^ *p-*value		0.070	0.064	0.122	0.037	0.012
CSFP < 250 mm H_2_O	38	16.5 (8.2/27.1)	21.8 (14.5/27.3)	28.6 (22.8/37.9)	0.80 (0.42/1.14)	0.50 (0.32/0.66)
CSFP ≥ 250 mm H_2_O	12	18.6 (16.6/25.6)	25.4 (21.1/30.0)	34.6 (28.1/38.9)	0.34 (0.24/0.52)	0.26 (0.19/0.38)
^†^ *p-*value		0.357	>0.999	0.183	0.024	0.037
Dehiscence	22	17.7 (12.1/24.1)	26.2 (18.3/29.3)	30.7 (24.8/37.9)	0.42 (0.29/0.62)	0.33 (0.23/0.41)
Diverticulum	24	23.7 (10.4/30.0)	21.3 (14.4/27.9)	31.7 (21.9/38.8)	0.88 (0.46/1.17)	0.53 (0.33/0.72)
Non-SSWAs	4	9.9 (6.1/13.9)	25.2 (24.3/27.8)	18.9 (14.4/21.3)	0.83 (0.61/1.62)	0.54 (0.4/0.79)
^‡^Intracohort Comparison *p-*value		0.183	0.628	0.190	0.026	0.035

† Continuous data measured using Mann-Whitney test.

‡ Continuous data measured using Kruskal Wallis test.

Vol_flow_ indicates flow volume, V_mn_ indicates mean velocity, V_max_ indicates peak velocity, PI indicates pulsatility index, and RI indicates resistive index.

Variables are expressed as mean ± standard deviation or median and interquartile range.

**Table 5:** Jugular hemodynamics of 50 venous PT subjects and 50 healthy controls.

	**Number of Cases**	**Bilateral total Vol_flow_**	**Bilateral averaged V_mn_**	**Bilateral averaged V_max_**	**Bilateral averaged PI**	**Bilateral averaged RI**
		**g/s**	**cm/s**	**cm/s**	**–**	**–**
Venous PT population	50	18.0 (9.0/26.9)	22.0 (15.9/28.1)	30.7 (23.6/38.4)	0.62 (0.34/1.06)	0.41 (0.26/0.64)
Normal population	50	19.9 (14.0/25.6)	23.2 (19.4/31.1)	33.9 (26.2/46.8)	0.87 (0.57/1.15)	0.57 (0.42/0.69)
*p* value^†^		0.346	0.262	0.126	0.047	0.016

† Continuous data measured using Mann-Whitney test.

Vol_flow_ indicates flow volume, V_mn_ indicates mean velocity, V_max_ indicates peak velocity, PI indicates pulsatility index, and RI indicates resistive index.

Variables are expressed as median and interquartile range.

In comparison to controls, there was a significant difference found in bilateral-averaged PI and RI between the case and control groups (bilateral-averaged PI: Mann-Whitney test, *p* = 0.047; bilateral-averaged RI: Mann-Whitney test, *p* = 0.016). Other hemodynamic parameters were found insignificant between case and control groups (Table 5).

### Correlation among ITSS, BMI, jugular hemodynamics, and CSFP

The ITSS score positively correlated with CSFP (Pearson's correlation test, *p* < 0.01, *r* = 0.476). Bilateral total Vol_flow_ (Spearman correlation coefficient = 0.326, *p* = 0.02), bilateral averaged PI (Spearman correlation coefficient = - 0.383, *p* < 0.01), and bilateral averaged RI (Spearman correlation coefficient = - 0.383, *p* < 0.01) were correlated with CSFP. To examine the impact of TS stenosis on the jugular outflow, we found that the normalized degree of ipsilateral TS stenosis was positively correlated with ipsilateral Vol_flow_ (Spearman correlation test, *p* = 0.010, *r* = 0.373).

**Figure 4:**
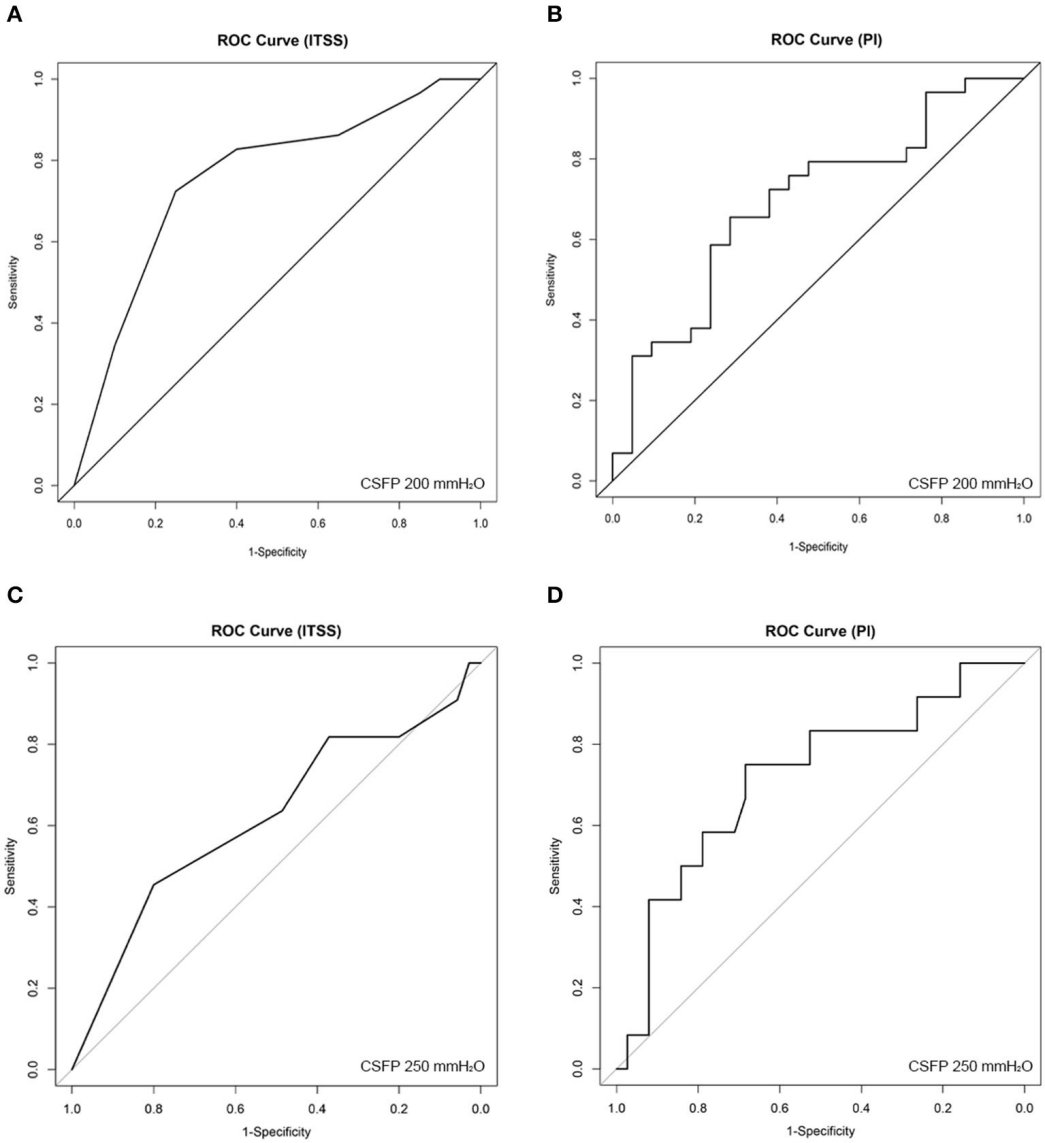
Receiver operating characteristic (ROC) curve analysis for **(A)** (200 mmH_2_O threshold) index of transverse sinus stenosis (ITSS), **(B)** (200 mmH_2_O threshold) bilateral-averaged pulsatility index (PI), **(C)** (250 mmH_2_O threshold) ITSS, and **(D)** (250 mmH_2_O threshold) bilateral-averaged PI.

According to multivariate linear regression using the stepwise method, ITSS and bilateral averaged PI were linearly correlated with CSFP, resulting in the following equation: *Y*_CSFP_ = 6.543 *X*_ITSS_ – 18.028 *X*_PI_ + 3.811 *X*_BMI_ (*R* = 0.611, *R*^2^ = 0.373, *p* < 0.01). ROC analysis showed that for 200 mmH_2_O threshold, the area under the ROC curve of ITSS was 0.757, and the best ITSS cut-off value was 8.5 (*p* = 0.002, 95% CI = 0.616–0.898) with a sensitivity of 72.4% and specificity of 75.0%; for 250 mmH_2_O threshold, ROC analysis was insignificant (*p* = 0.236). Additionally, ROC analysis showed that for 200 mmH_2_O threshold, the area under the ROC curve of PI was 0.693, and the best PI cut-off value was 0.467 (*p* = 0.021, 95% CI = 0.544–0.842) with a sensitivity of 65.7% and specificity of 81.8%; for 250 mmH_2_O threshold, the area under the ROC curve of PI was 0.718, and the best PI cut-off value was 0.467 (*p* = 0.024, 95% CI = 0.548–0.888) with a sensitivity of 68.4% and specificity of 75.0% (Figure 4).

## Discussion

### Characteristics of CSFP in subjects with venous PT as the solitary symptom

This is the first case series intended to screen the level of intracranial pressure among subjects with venous PT as the solitary symptom. CSFP ≥ 200 mmH_2_O threshold was found in 31 of 50 (62%) study subjects, and notably, 11 (22%) subjects whose CSFP surpassed a higher 250 mmH_2_O threshold. It is plausible that some degree of dysregulation of CSF exists among patients with venous PT as the only presenting symptom possibly due to TS stenosis, yet the level of CSFP may be insufficient to induce neurologic and ophthalmologic symptoms apart from PT. Given that BMI positively correlates with CSFP alongside intravenous pressure and trans-stenosis pressure gradient (52), we hypothesize that (1) the formation of SSWAs (possibly as a result of autoregulated compensatory mechanism to govern the increased central venous pressure) is vulnerable to the elevation of CSFP, and (2) the level of CSFP is likely limited to BMI and individual hydrodynamic conditions. Put differently, dysregulation of CSF homeostasis and sinovenous pressure affect subjects with PT induced by SSWAs.

CSFP and the degree of TS stenosis were not significantly different between the dehiscence and diverticulum groups. However, the average CSFP in the diverticulum group was lower than that in the dehiscence group. With the Monro-Kellie doctrine in mind, CSFP likely drops as the diverticulum expand outward (increase in the venous pool volume). However, it is noteworthy that elevated CSFP has been associated with clinical entities related to abnormal bone remodeling, such as CSF otorrhea/rhinorrhea and superior semicircular canal dehiscence (53–56), which are indirectly relevant to TS flow impact. Furthermore, SSWAs also exist in patients with normal CSFP and a high degree of TS stenosis. Thus, it remains challenging to elucidate (1) the cause of sigmoid plate erosion, whether by increased CSFP or TS stenosis, or a combination thereof, and (2) if subjects with SSWAs and disparate CSFP levels share the same underlying sigmoid plate erosion process that is underpinned by a mild to moderate increase in CSFP due to CSF-sinovenous dysregulation.

### Correlation between bilateral TS stenosis/contralateral TS hypoplasia and CSFP

Although CSFP in 19 (38%) subjects in this study were below 200 mmH_2_O threshold, the median ITSS score of 6 in these subjects was comparatively higher than that in the normal general population (44). Carvalho et al. found that ITSS ≥ 4 had a sensitivity and specificity of 94.7% and 93.5% for the diagnosis of CSFP ≥ 250 mmH_2_O, respectively (44). However, ITSS failed to predict 250 mmH_2_O CSFP threshold, possibly due to the limited number of cases with CSFP ≥ 250 mmH_2_O in this study. Moreover, a large case series also suggested that 200 mmH_2_O cut-off value is associated with TS stenosis, which is in line with our current findings (57). The best cutoff value of the ITSS score for discerning CSFP ≥ 200 mmH_2_O in the current study population was ≥ 8.5 with less sensitivity and specificity compared to Carvalho's study. Since the ITSS score was significantly higher in SSWAs groups than in the non-SSWA group, we deduce that the higher cut-off value and lower sensitivity/specificity of the ITSS score in this study resulted from the higher degree of bilateral TS stenosis with a mixture of normal/borderline CSFP in subjects with SSWAs and the use of normalized degree of stenosis to measure the cross-sectional area of TS lumen.

The prevalence of unilateral hypoplastic TS was found to be 20–39% in the general population (43, 58). In this study, CSFP was significantly higher in subjects with TS hypoplasia than in those without TS. Although the sinus wall collapses secondary to the elevation of CSFP, or *vice versa*, remains contentious (21, 24); it is plausible that the presence of TS hypoplasia in the current participants is innate, and subjects with unilateral hypoplastic/aplastic TS may be more susceptible to an increase in CSFP when some degree of stenosis exists in the dominant TS.

### Presence and impact of AG on CSFP

The growth of the AG was hypothesized to occur under long-term mechanical stress exerted by the intracranial CSFP on the surrounding bone-fibrous and vascular structures (30). The number of TS AGs discovered in our subjects was relatively close to the 45.5% of participants with IIH, as observed by Yu et al. (24). Although the pseudopodial anatomic structure (arachnoid membrane) of the AG can potentially occlude the TS lumen, invagination of the AG normally causes *in situ* filling defect of the venous flow rather than a circumscribed intrinsic TS stenosis, which is unlikely to deform because CSF resorption requires continuous positive pressure gradient between intracranial pressure and venous sinus pressure, and the positive pressure gradient should always surpass sinovenous intramural pressure (30). Based on Haraldsson et al. finding, the size of AG shrinks after CSF removal also indicates that the invagination of AG into TS lumen likely indicates abnormal CSF circulation (59). Nevertheless, most intrinsic TS stenosis in our study was presumably caused by sudden thinning of the TS lumen, resulting in a higher degree of luminal stenosis than that caused by a small AG, which obliquely reflects why the number of AG in TS was higher in the low CSFP group than high CSFP group. Additionally, brain herniation into the AG was found in only two subjects, none of whom were situated in the TS. Even though a low proportion of subjects presented with brain herniation into the AG, subjects with brain herniation into the AG need to be suspected of having elevated CSFP (33, 60).

### Reduced pulsatility of sinus outflow: An indicator for CSFP elevation

Jugular PI was negatively correlated with CSFP, with inferential significance based on the current multilinear regression analysis. In line with Unnerbäck et al., a decreased pulsatile component of the venous outflow was observed in subjects with increased CSFP using a venous pulsatile index measured at IJV using phase-contrast MR (39). However, MR methods tends to underestimate the magnitude of peak systolic velocity (61). Furthermore, PI and RI are dimensionless indices and reflect sufficient information on the pulsatility of the venous outflow when gaged at the upper IJV closer to the heart, thus requiring no amplification coefficient. This also explains why RI were significantly different between the case and control groups. Since global congestion of venous trees induce the augmentation of intramural venous pressure and pressure gradient, the siphoning of the venous return can be arrested owing to the ascent of streamwise resistance of *vis a tergo* and pressure external fluid, a hydrodynamic mechanism related to the Starling resistor (62). Additionally, we also found a moderate positive correlation between intracranial pressure and bilateral total Vol_flow_ congruous with Schoser et al. finding, suggesting that an increased cortical venous outflow likely displaces into the dural sinus compartment after a rise in CSFP (63). This notion is self-evident that flow velocity and volume reduced after CSFP was lowered. It is noteworthy, however, that bilateral total Vol_flow_ can be greatly impacted by interindividual hemodynamic and sinus morphological differences in alternative collateral venous and spinal compartments. To sum up with our findings, PI has more diagnostic value regarding patients' CSFP than other observed jugular hemodynamic parameters in this study.

In previous literature regarding PT, Li et al. found that PI measured at TS was higher in patients with venous PT than in normal controls, suggesting that the elevated PI was due to lowered vascular compliance (64). Contrary to their findings, jugular PI and RI were significantly lower than the normal controls in our study; the data discrepancy likely resulted from the measurement of different regions of interest. Furthermore, it is notable that jugular V_mn_ and V_max_ were also found to be insignificant between venous PT participants and controls, which differs from other studies that measured flow velocity in the TS region. We postulate that the stark differences result from the difference in the cross-sectional area of the dural venous sinus and IJV, which varies significantly segmentally among study subjects. Additionally, the high velocity jet flow or vortex may produce drastic difference in pulsatile component due to the fluctuation of the intravascular pressure, which can occasionally be observed at the upper IJV.

### Study limitations and further directions

This study had some limitations. The sample size of the non-SSWA group was relatively small, although this type of patient is less frequently encountered in otologic clinical practice. Second, we did not measure the size of the AG because the hemodynamic effect caused by an AG cannot be qualitatively assessed merely by planar measurements. Nevertheless, obstruction of the TS lumen caused by TS AG or TS septation falls into the category of TS stenosis, which was considered and compared in this study. The investigation of hemodynamic perturbation caused by AG invagination is warranted in our future studies. Furthermore, evaluating CSFP using uncorrelated variables, i.e., ITSS and jugular hemodynamic parameters, can avoid collinearity when executing multivariate linear regression analysis. Most TS stenoses in our subjects were not caused by TS variations, but by the sudden innate thinning of the TS sinus lumen. Additionally, measurement bias such as interobserver variability and the exclusion of collateral venous outflow drainage are major influential factors of jugular hemodynamics measurement, although to quantify the global venous network using Doppler ultrasound at a unified location is currently infeasible since venous branches are small and highly subject-independent. To that end, MR techniques may be a more practicable approach in acquiring the hemodynamic pattern of entire intracranial and extracranial venous system in contrast to regional planar Doppler measurements. Finally, further multicenter quantitative and qualitative studies to incorporate more morphologic and hemodynamic related variables are warranted to establish a predictive model for CSFP evaluation.

## Conclusion

Patients with venous PT as the only presenting symptom should be suspected to have borderline or mildly increased CSFP when the degree of bilateral TS stenosis and BMI are high and the PI of the jugular outflow is reduced. Since vascular anomalies commonly prevail in patients with PT, objective morphological and hemodynamic parameters such as ITSS and bilateral-averaged PI measured by jugular Doppler ultrasound can assist neuro-otologists in predicting the level of CSFP in individuals rapidly and non-invasively. Unless the endoluminal invagination of the AG obstructs TS outflow, the presence of AG in TS without encephalocele and the empty sellae are pathognomonic findings in determining the level of CSFP in patients with venous PT; these radiological signs likely indicate the impairment in dynamics of CSF resorption and sinovenous hemodynamics, which further quantitative hydrodynamic investigations are warranted to differentiate those with or without dysfunctional CSF reabsorption.

## Data availability statement

The original contributions presented in the study are included in the article/supplementary material, further inquiries can be directed to the corresponding author/s.

## Ethics statement

The studies involving human participants were reviewed and approved by Ethical Committees of the Eye, Ear, Nose, and Throat Hospital in Shanghai, China. The patients/participants provided their written informed consent to participate in this study.

## Author contributions

XG collected ultrasound data and drafted the manuscript. Y-LH provided major conceptual design, ran statistical analysis, and assisted in drafting the manuscript. SW performed the MR examinations. SS assisted in manuscript drafting. WW is the lead surgeon who supervised this study. All authors contributed to the article and approved the submitted version.

## Funding

This study was supported by the National Science Foundation of China (NSFC No. 81670933).

## Conflict of interest

The authors declare that the research was conducted in the absence of any commercial or financial relationships that could be construed as a potential conflict of interest.

## Publisher's note

All claims expressed in this article are solely those of the authors and do not necessarily represent those of their affiliated organizations, or those of the publisher, the editors and the reviewers. Any product that may be evaluated in this article, or claim that may be made by its manufacturer, is not guaranteed or endorsed by the publisher.
